# Covid-19 restriction policies and shopping streets

**DOI:** 10.1371/journal.pone.0267160

**Published:** 2022-07-29

**Authors:** Hans R. A. Koster, Jos van Ommeren, Cheng Keat Tang, Nander Bras

**Affiliations:** 1 Department of Spatial Economics, Vrije Universiteit Amsterdam, Amsterdam, Netherlands; 2 Center for Market Studies and Spatial Economics, Higher School of Economics University, Saint Petersburg, Russia; 3 Tinbergen Institute, Amsterdam, Netherlands; 4 Department of Economics, Nanyang Technological University, Singapore; University of Wisconsin Madison, UNITED STATES

## Abstract

Policymakers around the world are enforcing mobility restriction policies such as lockdowns, facemask requirements and social distancing to curb the spread of Covid-19. While these policies are effective in preventing the spread of virus, the economic implications are not well understood. We contribute to the literature by examining the impact of these policies on the offline retail sector. Specifically, we measure the effects of these policies on the daily number of shoppers passing by, which we refer to as ‘footfall’, along major shopping streets in Netherlands. We rely on unique proprietary Wifi data to accurately measure footfall. Our findings imply that all these policies attribute to a non-trivial reduction in footfall levels along shopping streets. While lockdowns led to a 50% reduction in footfall along major shopping streets, shopping streets faced with facemask regulations also experience a 25% drop in human traffic. A reduction in footfall translates into a substantial reduction in retail income of between 12% and 25%.

## Introduction

Covid-19 has caused unprecedented economic impacts from the implementation of mobility restriction policies to curb the spread of the pandemic. The existing literature has shown that the fear of infection and lockdowns have considerable impacts on retail spending [1–4], human mobility and shopping patterns [5]. Hence, one of the most affected sectors is the retail sector, which include retail shops, service providers (*e.g.* hair saloons, gyms), restaurants and bars. The productivity of retail activities is paramount for the functioning of the economy as this sector contributes around 8–10% of GDP. Shopping areas are considered by many as being important for the liveliness of cities [6–8]. Although online shopping is on the rise, it accounted for merely 10% of overall retail sales before the start of the pandemic [9]. While we readily purchase clothing and household items online, many still prefer the in-person experience from visiting restaurants and bars. This has clearly changed since the pandemic [5]. With the enforcement of mobility restrictions of citizens, online shopping has grown by about 80% [10], clearly illustrating the adverse impact of the pandemic on the offline retail sector.

**Fig 1:**
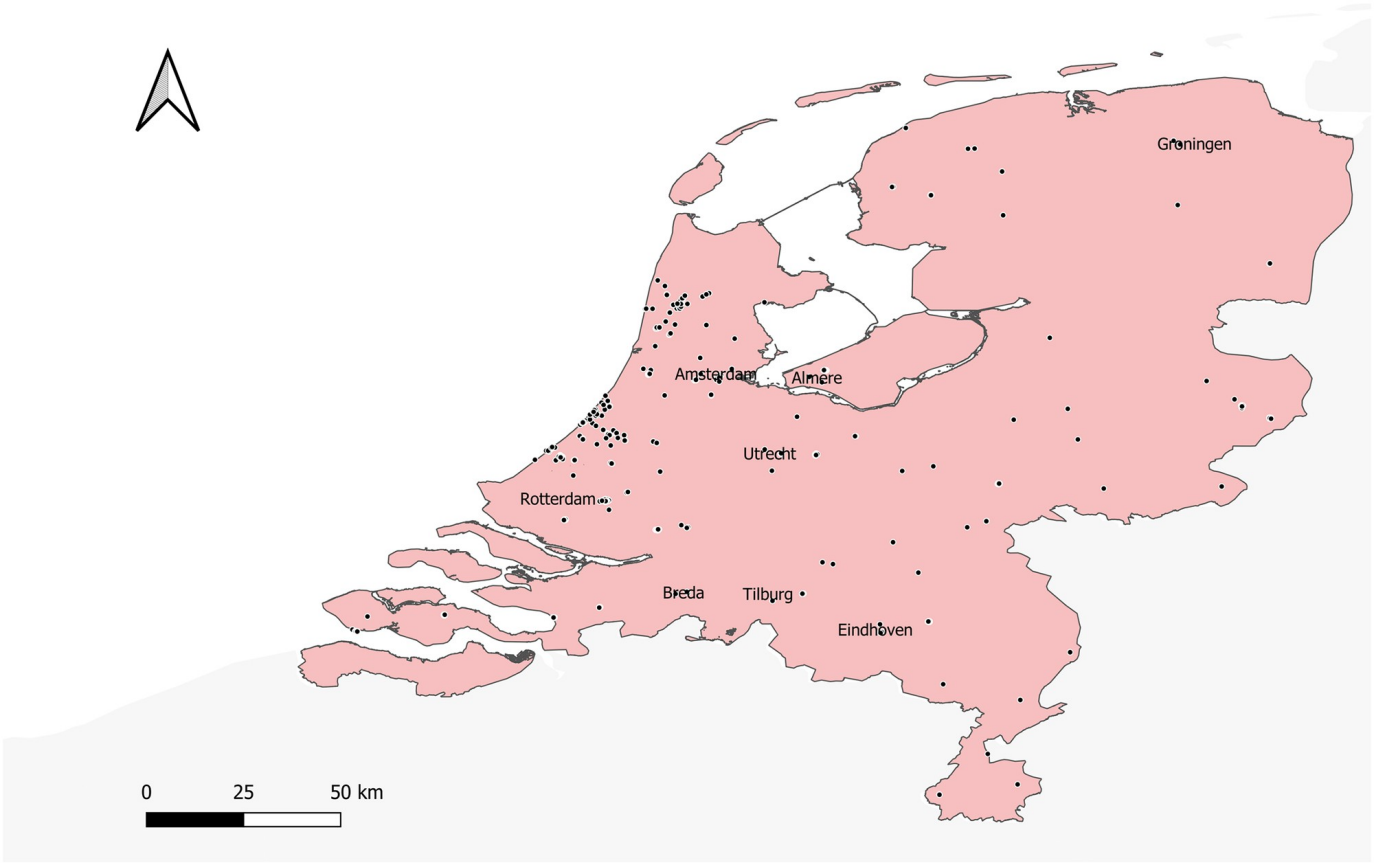
Spatial distribution of RMC sensors. The map is made with *Natural Earth* by the authors. The locations of footfall counters, denoted in dots, are provided by Bureau RMC.

This paper examines the effects of various Covid mobility restriction policies, such as lockdowns, and facemask regulations, on offline retail productivity in Netherlands. Specifically, we measure the impact of these policies on the footfall along shopping streets, which are particularly important in Europe as shopping malls are typically rare. We rely on unique proprietary real-time Wi-Fi data provided by Bureau RMC to measure daily footfall (or visitor flow) along major shopping streets for 530 locations across the Netherlands. Fig 1 presents the spatial distribution of RMC sensors across the Netherlands. Most sensors are located around city centers of major cities such as Amsterdam, Rotterdam, and The Hague. Our research strategy involves comparing footfall levels during treatment periods when restriction policies are enforced with non-treatment periods to quantify the impact of mobility restriction policies on footfall. Furthermore, with detailed information of the number and type of shops around each shopping street, we are able examine the impact of mobility restriction policies across space and for different type of shopping streets (*e.g.* with a high density of shops, or with a high share of clothing stores).

Footfall is an important measure for the attractiveness of a shopping location and is the main determinant of retail productivity [11]. Research has shown that a 1% increase in footfall corresponds to a 0.5% increase in rent, which is a common proxy for retail income (or productivity) [12]. The idea is that if shops sell more products at certain locations because of higher footfall, they are willing to pay higher rents, explaining higher rents in busy shopping streets. We estimate the effect of footfall on retail rents for the period between 2010 and 2020 and assume that the long-run effect of footfall on rent also holds during the Covid-19 crisis. Given this assumption, we are able to estimate the economic impact of Covid mobility restriction policies.

**Fig 2:**
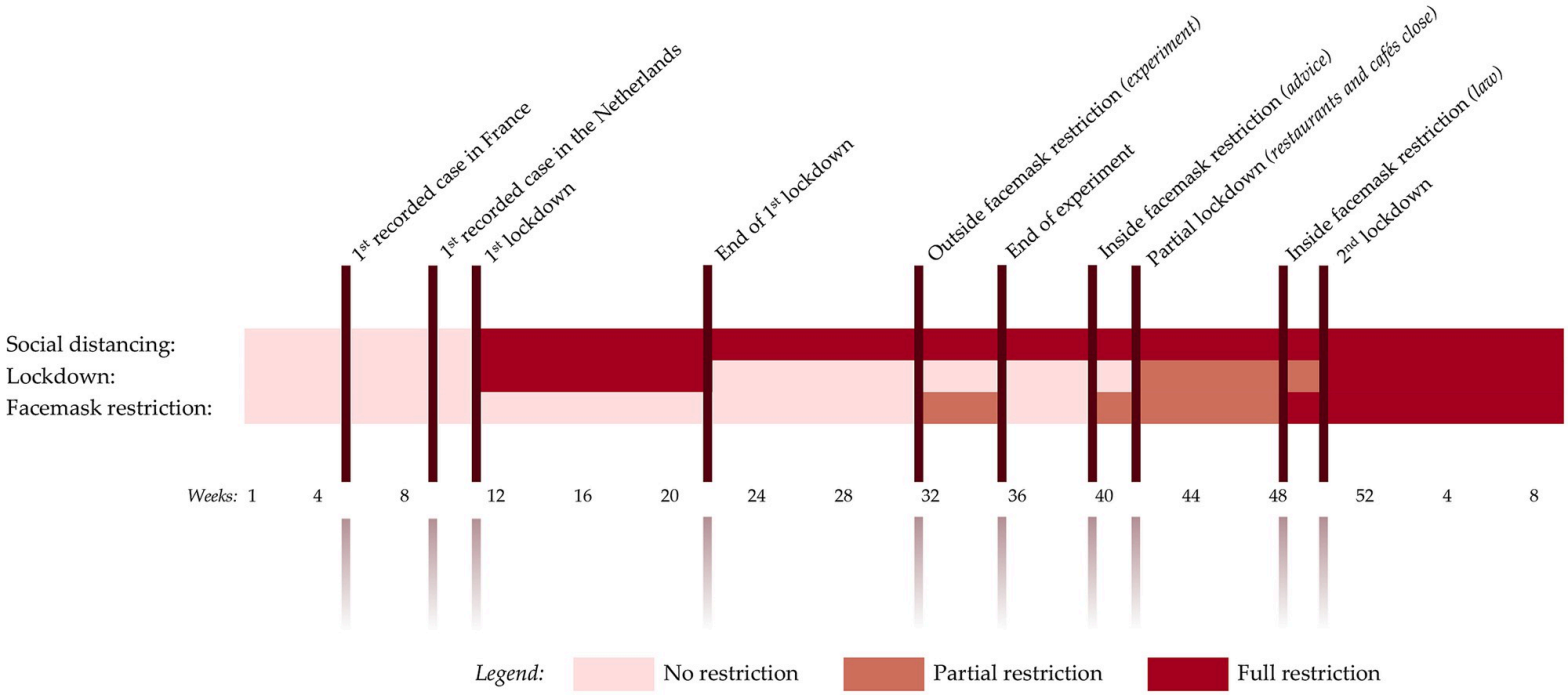
Timeline of Covid mobility restriction policies implemented in Netherlands.

We consider the effects of two different policies. The main policy implemented to curtail the spread of the virus is lockdowns. The first lockdown, which lasted for 3 months, was enacted on the 15^th^ of March and ended on the 1^st^ of June 2020. Although shops were allowed to operate, many shops were closed due to health concerns, lack of employees and demand [13]. The rebound of Covid cases led to the enforcement of partial lockdown on the 1^st^ of October 2020 in which food and beverage (FNB) stores were obliged to close. Subsequently, a more stringent second lockdown, which stipulated that all (offline) shops selling non-daily products were not allowed to operate, was implemented on the 15^th^ of December 2020. We expect footfall to fall substantially along shopping streets as residents are advised to stay at home during lockdowns. Dutch policy makers also mandate the wearing of facemasks *inside* shops as well as *outdoors*. A short-lived pilot policy was applied to several busy shopping streets in the two of the largest Dutch cities (*i.e.* Amsterdam and Rotterdam) on the 1^st^ of December 2020. The effect of mandatory facemask requirements on footfall of shopping streets is less clear. Policy makers further implemented social distancing on the 15^th^ of March 2020. Individuals from different households were advised to keep a 1.5m distance from each other at all times. Fig 2 provides a timeline of the different restriction policies introduced by the Dutch government to curtail the spread of the virus.

Overall, we record sizable reductions in footfall along major shopping streets in Netherlands after the mobility restriction policies are enforced. Specifically, we observe that human traffic fell by more than 50% after lockdowns are enforced. This effect is noticeably stronger along streets with more shops, suggesting that individuals are avoiding bustling streets to mitigate the risk of infection. A 6-month lockdown, as observed in the Netherlands, has led to a 11% reduction in yearly rental income for the retail sector. Further, footfall experienced a 25% drop in streets where mandatory outdoor facemask regulations are enforced. These latter reductions in human traffic translate into a 12% decrease in retail income in shopping streets.

We contribute to the existing literature on several fronts. First, we examine a wide variety of mobility restriction policies introduced by the Dutch government during the pandemic to understand the economic implications of mobility policies on the retail sector. Second, our study draw inferences from an extensive data set on daily footfall measures across Netherlands using a variety of rigorous empirical strategies to enhance both the internal and external validity of our findings. More broadly, our study contributes to the literature on retail productivity, showing that national retail policies have implications that vary strongly over locations [14–16]. The study also underscores the importance of footfall for retail productivity [12, 17].

## Materials and methods

We adopted a variety of empirical strategies to quantify the effects of lockdowns and facemask regulations on footfall, before estimating the elasticity of footfall on retail rents to understand the economic effects of these policies on the retail sector. Specifically, we adopted a temporal regression-discontinuity design to measure the short-run effects of lockdowns as those have an immediate effect on footfall, and a difference-in-difference strategy to measure the impacts of facemask regulations. Finally, we estimate the causal effect of footfall on retails rents using an instrumental variable regression strategy, exploiting the exogenous variation in footfall from the locations of historical cinemas. A summary of the different approaches and the description is provided in Table 1. We provide more details on each of these methods in the subsequent sections.

**Table 1:** An overview of empirical methods adopted.

Empirical relationship	Empirical strategy	Description
Lockdowns ⇒ footfall	Temporal RDD	Comparing change in daily footfall right before and after various lockdowns are enforced.
Facemask regulation ⇒ footfall	Local DID	Comparing changes in daily footfall between regulated and adjacent unregulated areas before and after facemask regulations are enforced.
Footfall ⇒ rents	IV	Estimating the elasticity of annual rents with respect to footfall, exploiting exogenous variation in footfall from historical cinemas.

### Lockdowns

We exploit the temporal variation in footfall to capture the effects from the implementation of various lockdown events, labelled by the subscript *n*, on log footfall across the Netherlands. Here, we examine the effects of the start of the 1^st^ lockdown (on the 15^th^ of March 2020), 2^nd^ lockdown (on the 15^th^ of December 2020). Hence, *n* = 1, 2. We start by estimating the following temporal regression-discontinuity model:

$$\begin{array}{c} \text{ln}\left(F_{it}\right)=\alpha_{i}+\sum_{n}\gamma_{n}L_{nt}+f_{n}\left(D_{it}\right)+X_{it}^{\prime }\delta +\epsilon_{int}, \end{array}$$ (1)

where the dependent variable, ln(*F*_*it*_), is the natural logarithm of footfall recorded by RMC sensor *i* on day *t*. Our key variable of interest is *L*_*nt*_, which is a binary variable that takes the value of 1 at day *t* after type *n* lockdown is enforced. Hence, *γ*_*n*_ captures (approximately) the percentage change in footfall across all RMC sensors from type *n* lockdown. *f*_*n*_(*D*_*it*_) is a polynomial function of the number of days from the lockdown event *n* and we allow these trends of footfall to vary before and after the enforcement by interacting these polynomials with *L*_*nt*_. We allow footfall trends to vary flexibly by including second-order polynomials to ensure that the discontinuous jumps in footfall around the lockdown events are not driven by pre or post event trends in footfall. *α*_*i*_ denotes RMC sensor fixed effects that partial-out time-invariant unobserved differences between locations. We further control for public and school holidays, and weather conditions. These control variables are captured by *X*_*it*_. To mitigate bias from unobserved time shocks, we constrain our analysis to observations within a window of 90 days around the type *n* lockdown. Put differently, we are comparing the sharp changes of footfall before and after the lockdowns are implemented to capture the causal effect of lockdowns on footfall. This empirical strategy is meaningful because lockdowns have an immediate and sharp impact on the mobility of shoppers.

We are also interested to examine whether effects of lockdown policies differ systematically across locations, as captured by shopping street characteristics that include shop density and share of shop type. Specifically, we count the number of shops within 500 meters from the shopping street to measure shop density, and we classify shops within 500 meters into three main categories—clothing, food and beverages (FNB) and other retail shops—before computing the share of the different shop types. We allow the effects of lockdown to vary across locations by interacting *L*_*nt*_ with these shopping street characteristics. These estimates are meaningful as they capture the efficacy of lockdowns across different locations that vary by street characteristics.

Initially, we treat the interaction term with shop density as exogenous. However, one may argue that the shop count could be correlated with unobserved factors that affect footfall. Moreover, there could be concerns of reverse causality as retail firms are more likely to be situated at locations with high footfall. To deal with potential endogeneity concerns, we instrument shop density with the number of cinemas in 1930 within 500m from the location of the sensor. Historically, cinemas were small with one screen only and were often located along shopping streets. The identification assumption is that the historical number of cinemas are correlated with the current shop density (instrument relevance) and are only affecting footfall levels through shop density (exclusion restriction). For a fuller discussion on the validity of the instrument, we refer to [12]. We compute Kleibergen-Paap *F*-statistic to illustrate instrument relevance. The results from the estimation of Eq (1) are presented in Panel A of Table 2.

**Table 2:** The impact of lockdowns on footfall.

	(1)	(2)	(3)
**Policy**	-0.635^a^	-0.624^a^	-0.616^a^
	(0.078)	(0.071)	(0.071)
**Policy × Log Shop Counts**		-0.157^a^	-0.369^a^
		(0.049)	(0.111)
**Policy × Share of Other Retail Shops**		-0.782^c^	0.184
		(0.433)	(0.721)
**Policy × Share of Clothing Shops**		-1.215^c^	1.503
		(0.706)	(1.541)
**Policy × Share of FNB shops**		-1.005^b^	-0.030
		(0.434)	(0.759)
Obs	91822	90773	90773
Adj R2	0.79	0.78	0.36
Kleibergen-Paap F statistic			15.05

The dependent variable is log footfall. This table reports estimates from regression discontinuity design (RDD) on the impact of lockdowns on footfall that controls for public and school holidays, weather conditions, RMC fixed effects and second order polynomial time trends (quadratic). We limit our analysis to observations 90 days before and after various lockdowns. We exclude all days in which other mobility restriction policies are enforced. We instrument log shop counts with cinemas in 1930 in column 3. Two-way clustered standard errors at postcode and date levels are reported in parentheses.

^c^ p<0.10,

^b^ p<0.05,

^a^ p<0.01.

#### Facemask regulations

We measure the impact of mandatory facemask wearing outdoors on footfall (*F*_*it*_) using a local difference-in-difference approach. This policy was introduced in a limited number of dense shopping streets in Amsterdam and Rotterdam for a short period of time. We estimate the following specification:

$$\begin{array}{c} \text{ln}\left(F_{it}\right)=\alpha_{i}+\zeta_{1}T_{it}+\zeta_{2}T_{it}^{0-500m}+\tau_{t}+\epsilon_{it},\;\;\text{if}\;d_{it}<d^{\text{max}} \end{array}$$ (2)

where *T*_*it*_ takes the value of 1 for RMC sensors where outdoor facemask wearing is compulsory between the 5^th^ and 30^th^ of August, 2020. The effect on log footfall due to mandatory facemask regulation is captured by *ζ*_1_. To examine spillover effects on neighboring shopping streets, we include $T_{it}^{0-500m}$, a dummy indicator with a value of 1 for RMC sensors within 500m from a regulated shopping street. The spillover effect is captured by *ζ*_2_. We expect that *ζ*_1_ < *ζ*_2_ < 0. Put differently, we are comparing changes in footfall before and after the facemask regulations are enforced between regulated and unregulated shopping streets and we allow the regulation to have spillover effects on areas within 500m. We include RMC sensor and *date* fixed effects, where the latter control for general trends in footfall across areas, and other time-varying variables, which are thought to be important such as public and school holidays and the number of Covid cases. Our identification hinges on the assumption that regulated and unregulated shopping streets have similar footfall trends. This assumption may not hold because regulated shopping streets are located around the city centers of the largest cities in the Netherlands. To address this, we restrict our sample to RMC sensors within 10km from the regulation boundary to ensure that the trends in footfall are comparable between regulated and unregulated areas (hence, we set *d*^max^ = 10). We further show the sensitivity of the results by restricting the sample to unregulated areas not more than 1km from regulated areas. Estimates from Eq (2) are plotted in Fig 5.

### The effect of footfall on rent

We estimate the effect of footfall on rent relying on two additional data sources, Strabo and Vastgoeddata, that provides information on commercial retail rents, size and construction year for 966 retail establishments close to RMC points (within 100m) from 2010 to 2020. We also extend footfall data from RMC back to 2010 to estimate the relationship between footfall and retail rents. Our estimation equation takes the following form:

$$\begin{array}{c} \text{ln}\left(p_{ijt}\right)=\alpha \text{ln}\left(F_{ijt}\right)+\beta x_{ijt}+\eta_{j}+\theta_{t}+\epsilon_{ijt}, \end{array}$$ (3)

where *p*_*ijt*_ be the rent paid by retail firm *i* in shopping district *j* in year *t*, which is a function of footfall (*F*_*ijt*_). *x*_*ijt*_ denotes shop and location characteristics (*e.g.* shop size, construction year, etc.). The key parameter of interest, *α*, captures the corresponding percentage change in rents from a 1% change in footfall. *η*_*j*_ are district fixed effects, *θ*_*t*_ are year fixed effects and *ϵ*_*ijt*_ is a random error term. Because our dataset is not very large, we cannot include the detailed shopping street fixed effects as in [12, 18], but rely on slightly more aggregate district fixed effects. We further employ an instrumental variable strategy as there are concerns whether footfall is correlated to unobserved locational characteristics that could affect rents. Hence, we instrument footfall with the number of cinemas in 1930 <200m. Historically, most cinemas were small with one screen only, and were located in shopping streets. Hence, the buildings hosting these cinemas were not very different from the surrounding buildings. The buildings of the closed cinemas from the 1930s are now frequently used as shops, but also attract other businesses. We further control for the number of cinemas in the vicinity in 2010 to address the issue that the locations of current cinemas may be correlated to cinemas in the past. The main identifying assumption when relying on long-lagged instruments is that past unobservable characteristics of either stores or locations are uncorrelated to current unobservables conditional on control variables, district and year fixed effects.

The estimates associated with Eq (3) are presented in Table 3. Finally, we rely on the elasticity of annual rents with respect to footfall (*α*) from Eq (3) and the effects of lockdowns on footfall (*γ*_*n*_) from Eq (1) to calculate the economic impacts of Covid mobility policies on the retail sector.

**Table 3:** The impact of footfall on retail rents.

	(1)	(2)	(3)
**Log of Footfall**	0.6291^c^	0.5145^a^	0.4864^b^
	(0.3701)	(0.1144)	(0.1902)
Property and location controls	Yes	Yes	Yes
District fixed effects	Yes	No	No
Municipality fixed effects	No	No	Yes
Year fixed effects	Yes	Yes	Yes
Obs	966	983	975
Kleibergen-Paap *F*-statistic	3.135	36.97	8.211

*Notes:* The dependent variable is the log of rent per m^2^. We instrument for footfall with the number of cinemas in 1930 and control for the number of cinemas in 2010. Property controls include the log of size of the property and 10 construction year decade dummies, while location controls include the number of busstops <200m, as well as the number of listed buildings <200m. Robust standard errors are clustered at the RCM scanner level and in parentheses.

^a^*p* < 0.01,

^b^*p* < 0.05,

^c^*p* < 0.10.

## Results

### Impacts of mobility restriction policies on footfall along shopping streets

**Fig 3:**
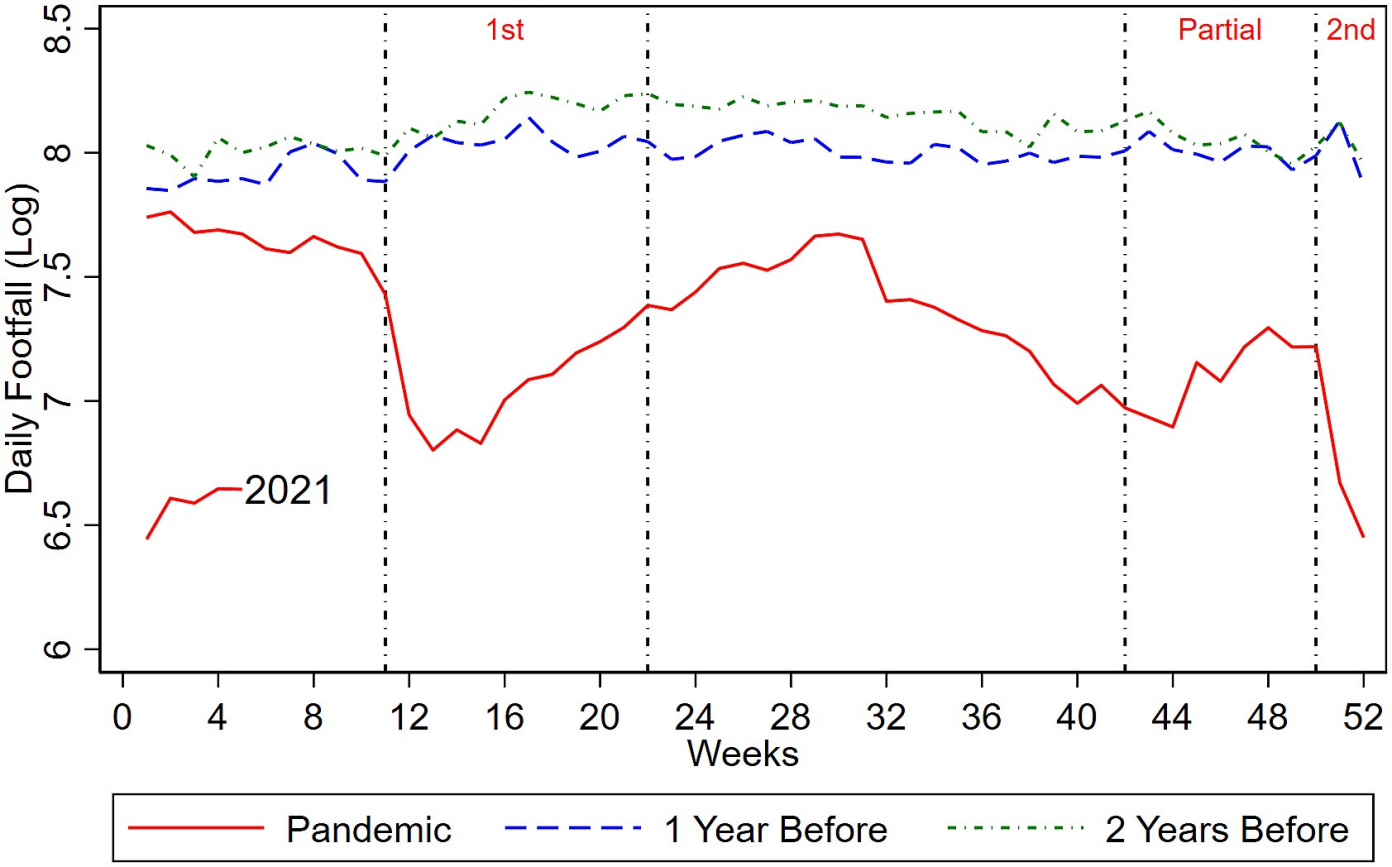
Footfall over time. The log of weekly footfall during first *(1^st^)*, partial *(Partial)* and second lockdown *(2^nd^)* in the year of pandemic, 1 year before and 2 years before.

Fig 3 plots the average weekly footfall levels along shopping streets from 2018 to 2021. We record sizable drops in footfall after the first lockdown was implemented before a slight upward rebound when the lockdown was relaxed. While the enforcement of partial lockdowns did not matter much, footfall dipped sharply after the second lockdown was implemented. We also observe a slight reduction in footfall just before the first lockdown, consistent with the notion that the residents are fearful of Covid infection [5]. We no longer observe a pre-lockdown dip in footfall before the second lockdown was implemented. This could be due to the fact that residents are more knowledgeable and aware of the virus one year into the pandemic.

#### Lockdowns

**Fig 4:**
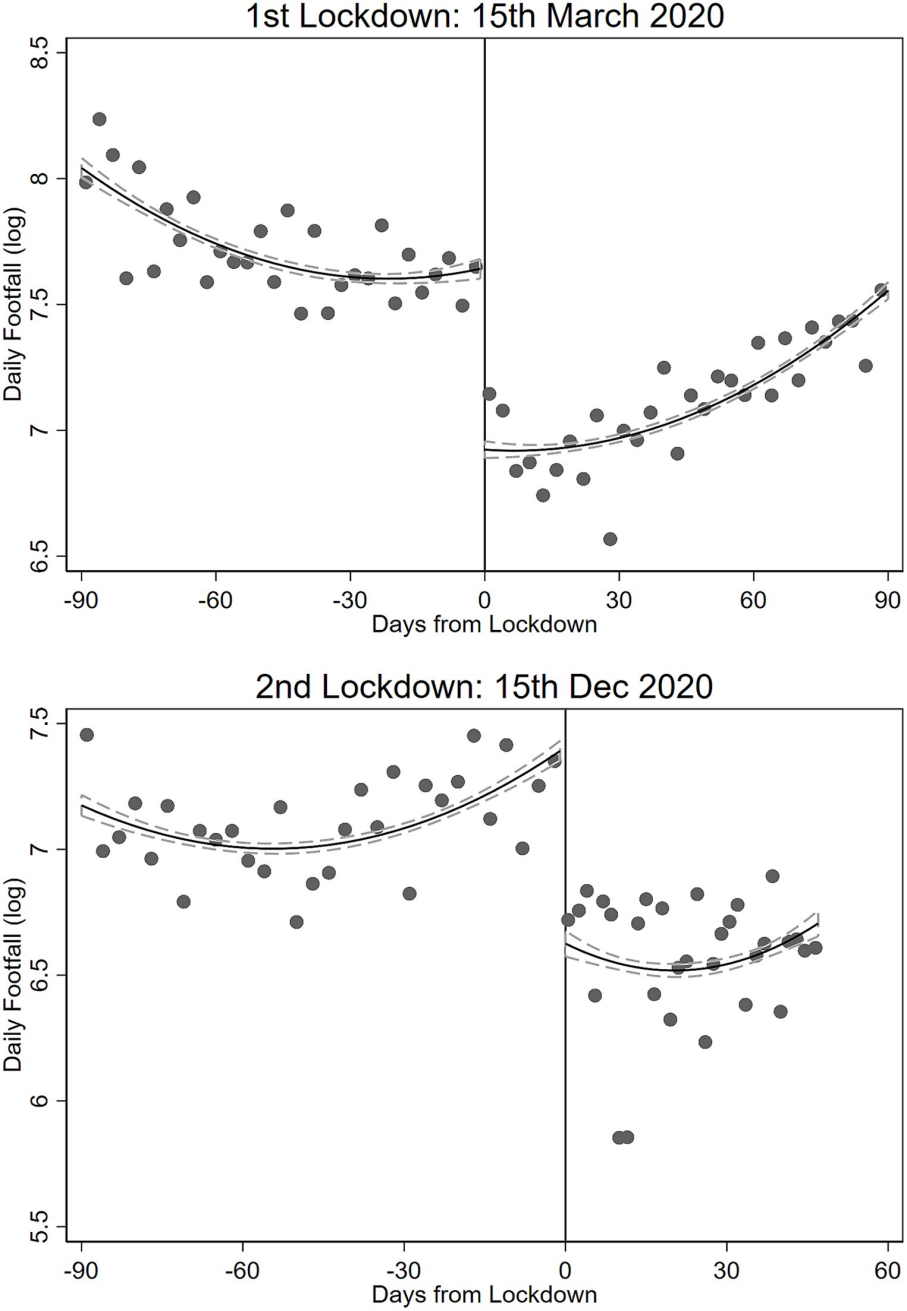
Lockdowns and footfall. We show log daily footfall 90 days before and after first and second lockdown. We control for a second-order polynomial on both sides of the threshold. Dashed line denotes 95% confidence interval.

Fig 4 provides graphical evidence of the impact of the first and second lockdown on footfall by plotting smoothed residuals of average log footfall levels right before and after the implementation of the first and second lockdown after controlling for second-order polynomial of time trends on both sides of the threshold. We document a sharp short run reduction in footfall at around 0.6–0.7 log points (or around 45–50%) around both events. One major concern is that the first lockdown is capturing the effects of both lockdown and social distancing (see Fig 2) as both policies are implemented simultaneously. Such concerns are mitigated for the second lockdown because social distancing is already in effect when the second lockdown was enforced. Hence, we can isolate the effect of social distancing by comparing the estimates from the first and second lockdown. Although there are slight differences in the stringency of these two events, graphical evidences from Fig 4 indicate that the impacts from the two lockdowns are virtually the same, suggesting that social distancing has a negligible impact on footfall in the short run. This is unsurprising because social distancing is unlikely to be as impactful as lockdowns because it is merely an advice to the public to keep a safe distance from others. Furthermore, social distancing is likely a slowly changing process that takes time to take effect. We provide additional analyses in the supplementary materials that control for higher-order polynomials of the time trend on both sides of the threshold and also examine the impact of partial lockdowns. Overall, we do not report discernible differences from Fig 4 after controlling flexibly for the time trend, and we do not report any effects associated with partial lockdowns.

Panel A of Table 2 reports average effect of the first and second lockdowns on footfall from a regression-discontinuity strategy that compares footfall right before and after the lockdowns are enforced (we refer to the Methods section for an extended discussion). Hence, we identify short-run effects of lockdowns. Overall, we record substantial drops in human traffic along shopping streets after lockdowns are enforced. Results from column (1) show that lockdowns implied a substantial 47% (exp(−0.635)−1 ≈ −47%) reduction in footfall. Our estimates indicate that lockdowns are very effective in curbing human flow, which differ from the results shown by [5] for US. They show that individual choices, because of fear of infection, played a much larger role in reducing human flow as substantial voluntary reductions in footfall were observed even before the lockdowns were enforced. Conversely, we do not observe a significant reduction in footfall before the lockdowns were enforced in Netherlands (see Fig 3), suggesting that fear did not play a significant role. The disparity in results between the two countries warrants more attention. This could attribute from the smaller economic consequences of being infected by Covid as Dutch have mandatory health insurance and short-term absenteeism from work due to Covid does not result in wage cuts. Furthermore, Dutch are less fearful of shopping along outside shopping streets during the pandemic as there are very few shopping malls in Netherlands.

In column (2), we further examine the heterogeneous effects of lockdowns depending on shop density and shop types. We observe that stronger lockdown effects along dense shopping streets. Footfall in the densest shopping streets (with a density that is two log points above the average, which is about two standard deviations) is 67% lower after the lockdowns are enforced. Conversely, we document imprecise and moderate differences of lockdowns across different shop types. There is suggestive evidence that shopping streets populated with clothing shops are more adversely affected by the lockdowns. For instance, for shopping streets with the largest share of clothing stores (around 40%), which is 20% above the mean, the effect of lockdowns is around exp(−1.215 × 0.20 − 0.624) − 1 ≈ − 58%. We further adopt an instrumental variable approach to estimate the causal effect of shop density on the effects of lockdown on footfall, instrumenting shop density with number of cinemas in 1930 in column (3). The idea here is that there could be unobserved factors that could be correlated with lockdowns and directly affect footfall. These results now indicate that shop types no longer matter, but the density effect is even more pronounced, reinforcing the finding that lockdowns affect shopping streets with denser cluster of shops.

#### Facemask regulations

**Fig 5:**
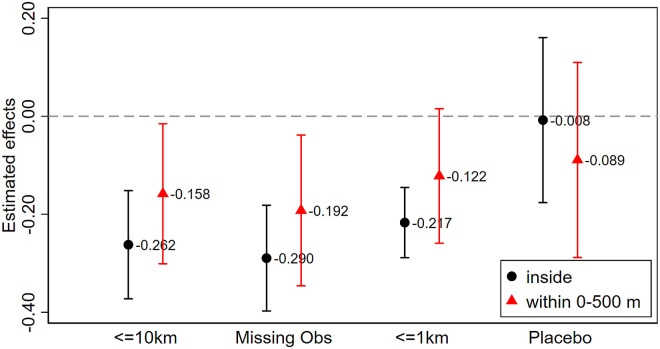
Facemask regulations and footfall. Dependent variable is the log footfall. Inside denotes regulated shopping streets and within 0–500m denotes unregulated shopping streets within 500m from regulated shopping streets. All regressions include RMC and date fixed effects. Tails denote 95% confidence intervals constructed from two-way clustered standard at postcode and date levels.

Fig 5 summarizes the impact of facemask regulations on footfall. We first restrict our analysis on a sample of RMC sensors within 10km from regulated streets in Amsterdam and Rotterdam. Our estimates suggest that once outdoor facemask wearing is enforced, footfall changes by exp(−0.262)−1 ≈ −23%, corresponding to a reduction of about 2, 500 shoppers per day. These findings are consistent with the notion that mandatory facemask regulations reduce footfall if shoppers care more about the hassle to wear facemasks rather than the reduced probability of getting Covid. Footfall in shopping streets near regulated streets is also adversely affected by this regulation. Specifically, we document a smaller, but still substantial drop of 14.6% (exp(−0.158)−1) in footfall, which implies a reduction of around 1,000 shoppers. We further constrain our analysis to sensors that provide data for more than 50% of the time as we are concerned whether sensors with substantial missing data could have erroneously measured footfall levels. If anything, the effects appear slightly more pronounced. We further limit our analysis to RMC sensors within just 1km from the facemask regulation boundaries to ensure that RMC locations affected and unaffected by the regulation are more comparable. Although our sample is now substantially smaller, we document a robust 19.5% and 11.5% reduction in footfall in shopping streets where facemask are required and in shopping streets within 500m of the regulated streets, respectively. Finally, we move the regulation window one year before the actual regulation date as a form of placebo test. Of course, any observations post 2020 are omitted to prevent the Covid-19 from driving our estimates. Here, we observe no statistically significant changes in footfall during the placebo period, suggesting that our results are unlikely to be spurious.

#### Estimating economic impacts measured by rent

So far our results have shown that lockdowns and facemask regulations substantially reduces footfall along shopping streets. To be able to ascertain the economic impacts of these policies on retail markets, we need an estimate of footfall on retail productivity. Following existing literature, we use retail rents as a proxy for retail income, which is an intuitive measure as retail firms are willing to pay higher rents at more productive locations [12, 17, 19]. We assume that the long-run effect of footfall on rent holds under the Covid-19 crisis. The economic impact of Covid policies can then be derived given the estimated elasticity of retail income with respect to footfall.

We report the relationship between footfall and retail rents in Table 3. In column (1), we instrument for footfall with the number of cinemas in 1930 within 200m of the property, while controlling for the number of cinemas in 2010. The elasticity is sizable and about 0.60. This estimate has two issues: the instrument is weak (*i.e.* the first stage *F*-statistic is substantially below the required rule-of-thumb of 10) and the estimate is only marginally statistically significant. To address both issues, we remove the district fixed effects in column (2). The point estimate is now about 0.50, and highly statistically significant. In column (3) we include municipality fixed effects rather than district fixed effects, but the results remain virtually unchanged. As in [12, 18] an elasticity of 0.50 implies that doubling footfall leads to rents that are 35% higher.

## Discussion

Our preferred estimate of the effect of footfall on rent from column (3) is around 0.50, which is substantially less than one [12]. The latter is important as it implies that the rental income losses from the reduction in footfall are less than proportional to the reduction in footfall. Specifically, these estimates suggest that rental income losses because of Covid-19 policies are approximately half the reduction in footfall, albeit still considerable. Using this estimate, we calculate the total negative effects of the lockdowns through reductions in footfall for (approximately) one year of Covid-19 policies for a representative shop in a shopping street. From March 15, 2020, until March 14, 2021, there were 168 lockdown days. We consider the preferred estimate to be the one reported in column (1) of Table 2. The loss due to these lockdown days is about 11% of annual rental income (≈ 0.50×(exp(−0.635) − 1) × (168/365)), which is certainly non-trivial.

These figures mask *extreme* differences between different shopping streets. It appears that dense shopping streets (usually with many clothing stores), bear the largest income losses. For example, the annual losses of lockdowns, despite that lockdowns were absent half of the time, are estimated to be about 16% (≈ 0.50 × (exp(−0.620 − 2 × 0.265)−1) × (168/365)) of rental income for shops located in dense shopping streets. On the other hand, losses for shops in low-density shopping streets are estimated to be about 2% (≈ 0.50×(exp(−0.620 + 2 × 0.265) − 1) × (168/365)) and are therefore barely noticeable.

Our focus is on the effects of Covid policies on the income of shops *through reductions in footfall*. Our estimates are silent about the increases in online sales on shops in shopping streets induced by Covid policies, which could compensate for the income losses induced by reductions in footfall. Given that the online sale increases are small as a percentage of overall sales (about 5–10%), combined with the plausible assumption that increases in online sales are not systematically related to shop location, our estimates can be essentially interpreted as the effect of Covid-19 policies on retail income including online sales.

## Conclusion

This paper carries three main messages. First, we show sizable economic costs of the different Covid-19 policies that restricted the mobility of the population to curb the spread of the virus through substantial reductions in footfall in shopping streets. We find large, persistent and heterogeneous effects of lockdowns, with an average reduction of 50% in footfall in Dutch shopping streets. A 6-month lockdown, as observed in the Netherlands, has led to a 11% reduction in yearly rental income for the retail sector. According to our estimates, the cost of the first and second lockdown in the Netherlands are of equal size though these lockdowns were quite different in nature over the number and types of shops that had to close. This makes sense as when a substantial number of shops closes, neighboring shops are adversely affected by the reduction in footfall.

Second, we observe that these policies are particularly effective in reducing footfall in *dense* shopping streets around city centres, in line with studies that show that *local* retail policies have strong spatial effects [20, 21]. Consequently, we observe that national retail policies have varying effects over locations [14–16]. This result is important not only because of the spatial implications of retail policies on rental income, but also, in the context of Covid-19, because the spread of the virus is more likely to occur along densely populated shopping streets. If these policies aim to reduce the spread of the virus through changes in shopping behavior in busy shopping streets, then these policies can be considered successful. Moreover, the observation that footfall reductions are much stronger in dense and busy shopping streets is evidence for the important role of shopping externalities in determining retail productivity.

Third, we observe that the obligation to wear facemasks *outdoors* in busy shopping streets induces not only sharp drop in footfall (with an average reduction of 25%), but also substantial reductions along adjacent shopping streets, showing that neighbouring shops are also indirectly affected [12, 18].

Findings from our study are likely to be relevant in the near future as countries continue to implement mobility restriction policies to control the pandemic as new variant of the virus emerges. As of August 2021, countries such as Australia, Austria, China, Malaysia, Singapore and the Netherlands have been re-introducing strict lockdowns again to curb with rising number of Covid cases. Hence, it is imperative to understand the economic implications of these policies to inform policy making.

It is also plausible that the pandemic has brought about long-term changes in offline retail, even when the pandemic is over, because of the increase in work from home arrangements and the reliance on online shopping platforms. Therefore, the substantial reductions in footfall we documented during the enforcement of Covid policies provide useful insights on what may happen to the retail sector in the long run.

## Supporting information

S1 File (ZIP)

S1 Data (ZIP)
