# Supplementary material for: Covid-19 restriction policies and shopping streets
Source: PLoS One. 2022 Jul 29;17(7):e0267160. doi: 10.1371/journal.pone.0267160 (PMC9337638; doi:10.1371/journal.pone.0267160)
Supplement: S1 File — (ZIP) [file pone.0267160.s001.zip › Supporting_Info.pdf]

Supporting information

The appendix reports auxiliary details and analyses to the main paper. We first provide more details and additional visualizations of the data used in the empirical analysis. We then report additional results and several robustness checks of our main specifications.

Data Description

RMC sensors

The RMC network consists of around 500 different Wi-Fi sensors distributed across shopping districts within the Netherlands. These sensors require an internet connection and access to electricity, which is provided by the shop where the sensor is placed. To comply with privacy regulation, shops need to give permission for the use of these sensors. Therefore, sensor placement is based on contracts with retailers, municipalities and retail associations. The sensor coverage of cities is heterogeneous, since certain municipalities have a contract for many sensors, while others have a contract for only one Wi-Fi sensor.

Summary statistics

Table S2 provides summary statistics for the entire sample of RMC sensors. There are, on average, around 434 shops within 500m from these RMC sensors. Around 20% of the shops sell clothing, 11% of the shops are for daily shopping (*e.g.* supermarkets, pharmacies), 26% of the shops are restaurants and bars etc., while the other 42% of shops refer to other retail types.

Table S3 further presents summary statistics associated with RMC sensors within the outdoor facemask regulated areas and unregulated areas. Shop profiles are quite different between regulated and unregulated facemask areas. There are a total of 23 RMC sensors within facemask regulated areas. Compared to areas outside, footfall levels are much higher within these regulated areas, suggesting that policy makers select populous streets for mandatory facemask wearing to prevent the spreading of the virus. These areas are typically closer to the city center, are more densely populated with shops and have a greater share of food and beverage shops compared to unregulated areas. We further constraint our analysis to areas 0 to 500m, 500 to 1000m and 1 to 5km from the regulation boundaries. Here, we observe that the daily footfall and shop profiles are more comparable for areas right outside (0-500m) but footfall is still considerably lower than those reported in regulated areas.

Finally, in Table S4, we present the mean levels of footfall and hospital admissions due to Covid-19 90 days before and after the first, second and partial lockdowns. Consistent with the main findings of this paper, we observe a stark decrease of around 46% in footfall after the first and second lockdowns are implemented. The relative effects on footfall are quite similar between the first and second lockdown, while the impact of the partial lockdown is negligible. It is also evident that the lockdowns are stop-gap measures strategically implemented to curb the sudden surge of cases. We observe a surge in the number of Covid-19 hospital admissions right after the lockdowns are enforced.

Variable definitions

Table S1. Variable definitions

Variable Name	Definition	Source
Daily Footfall	Absolute footfall counts captured by RMC sensor i on date t	RMC
Retail Rents	Rents per sqm paid by retail firm i in year t	Strabo
Covid 19 Hospital Admissions	Total number of Covid-19 hospital admissions on date t	Dutch agency of public health (RIVM)
Number of Shops	Total number of shops within 500m from RMC sensor i	Locatus
Share of Daily Shops	Number of Daily Shops divided by Total Number of shops within 500m from RMC sensor i	Locatus
Share of FNB Shops	Number of FNB shops divided by Total Number of shops within 500m from RMC sensor i	Locatus
Share of Clothing Shops	Number of Clothing shops divided by Total Number of shops within 500m from RMC sensor i	Locatus
Share of Other Retail Shops	Number of Other Retail shops divided by Total Number of shops within 500m from RMC sensor i	Locatus
Number of Historical Cinemas (in 1930)	Total number of historical cinemas (in 1930) within 500m from RMC sensor i	Locatus
Daily Rainfall	Daily amount of rainfall (in 0.1mm) recorded on date t	Dutch agency of meteorology (KNMI)
Mean Temperature	Average temperature (in Celsius) recorded on date t	Dutch agency of meteorology (KNMI)
Minimum Temperature	Minimum temperature (in Celsius) recorded on date t	Dutch agency of meteorology (KNMI)
Maximum Temperature	Maximum temperature (in Celsius) recorded on date t	Dutch agency of meteorology (KNMI)
Wind Speed	Average daily wind speed (in 0.1 m/s) recorded on date t	Dutch agency of meteorology (KNMI)
Sunshine	Number of Hours with Sunshine recorded on date t	Dutch agency of meteorology (KNMI)

Table S2. Descriptive statistics of shops within 500m of RMC sensors

	Mean	Std. Dev	Median	Min	Max
Number of Shops	433.95	331.17	355.00	2.00	1622.00
Number of Shops (Log)	5.69	1.03	5.87	0.69	7.39
Share of Daily Shopping shops	0.11	0.06	0.09	0.00	0.35
Share of Clothing shops	0.20	0.09	0.23	0.00	0.39
Share of Food and Beverage (FNB) shops	0.26	0.11	0.24	0.01	1.00
Share of Other Retail shops	0.42	0.13	0.43	0.00	0.94

Table S3. Descriptive statistics for outdoor facemask zones

	Regulated	Unregulated	0-500m	500-1000m	1-5km
Daily Footfall	11282.44<br>(75.77)	3624.33<br>(9.93)	5773.15<br>(43.23)	4018.30<br>(32.33)	4010.46<br>(42.54)
Daily Footfall (Log)	8.81<br>(0.01)	7.64<br>(0.00)	8.25<br>(0.01)	8.21<br>(0.01)	7.90<br>(0.01)
Number of Shops	961.32<br>(2.27)	392.60<br>(0.55)	677.47<br>(1.57)	449.93<br>(1.57)	252.14<br>(0.68)
Share of Daily Shopping shops	0.12<br>(0.00)	0.11<br>(0.00)	0.11<br>(0.00)	0.11<br>(0.00)	0.17<br>(0.00)
Share of Clothing shops	0.28<br>(0.00)	0.20<br>(0.00)	0.24<br>(0.00)	0.21<br>(0.00)	0.24<br>(0.00)
Share of Food and Beverage (FNB) shops	0.32<br>(0.00)	0.26<br>(0.00)	0.34<br>(0.00)	0.37<br>(0.00)	0.19<br>(0.00)
Share of Other Retail shops	0.28<br>(0.00)	0.43<br>(0.00)	0.31<br>(0.00)	0.31<br>(0.00)	0.40<br>(0.00)
Obs	25510	274824	16137	2205	10939
RMC sensors	23	312	16	2	10

Notes: Mean and standard error of means for regulated areas and non-regulated areas but within 0-500m, 500-1000m and 1-5km from the face mask regulation areas.

Distance to the city center and shopping streets characteristics

Here we explore the associative relationship between distance to the city center and 'structural' shopping street characteristics (shop type shares and shop density). We first determine the city center for all major cities. Then, for each RMC sensor, we calculate the distance to the nearest city center. While the average distance is 5km, most RMC sensors are within 25km from the city center. Table S5 reports the results of a regression of shopping street characteristics on distance (in kilometers), while including municipality fixed effects.

We find a strong association between distance to the city center and shop density. For a 1km increase to the city center, shop density decreases by 22%. Furthermore, the share of daily stores is considerably higher away from city centers. For a kilometer increase in distance to the city center, the share of daily shops decreases by 1.1 percentage point (about 10%). By contrast, the share of clothing stores is substantially higher in city centers. A 1km increase in distance to the center, decreases the share of clothing stores by 1.2 percentage points, so approximately 6%. For other shop shares we do not see a robust pattern.

Overall, in the Netherlands, the type of shops varies considerably with distance to the city center. Hence, as footfall affects the type of shops differently, Covid-19 policies also has a differential spatial impact within cities.

Additional graphical evidences

Figure S1 plots the natural logarithm of daily footfall 90 days before and after the facemask areas are designated for areas within the regulation boundaries, for areas outside, and for areas outside but within 0-500m from the regulation boundaries. Consistent with the regression results documented in Figure 5 (main manuscript), we document a larger drop in footfall in areas within the regulated areas compared to areas outside in terms of unconditional footfall. We also observe that footfall 0-500m outside are more comparable to the footfall within facemask regulation areas, justifying our

Table S4. Descriptive statistics before and after various lockdown events

	1st Lockdown		Partial		2nd Lockdown	
	**Before**	**After**	**Before**	**After**	**Before**	**After**
Daily Footfall	3230.09	1718.59	2448.00	2325.02	2234.21	1201.09
	(48.61)	(10.77)	(16.85)	(21.53)	(16.87)	(9.79)
Daily Footfall (Log)	7.58	7.07	7.33	7.12	7.11	6.57
	(0.01)	(0.01)	(0.01)	(0.01)	(0.01)	(0.01)
COVID-19 hospital admissions (Daily)	0.22	2.11	1.59	4.11	3.96	3.17
	(0.01)	(0.03)	(0.02)	(0.05)	(0.04)	(0.03)
Obs	5229	20350	26443	19626	28228	16159

Notes: Mean and standard error of means for footfall and Covid-19 admissions 90 days before and after the 1st, Partial and 2nd lockdowns are enforced.

Table S5. Distance to city center and shop counts and types

	(1) Log shop counts	(2) Share daily	(3) Share clothing	(4) Share FNB
Distance to the city centre	-0.2262[a]	0.0107[a]	-0.0119[a]	-0.0043
	(0.0305)	(0.0027)	(0.0039)	(0.0056)
Municipality fixed effects	Yes	Yes	Yes	Yes
Number of observations	502	502	502	502
R^2	0.5223	0.3610	0.4183	0.3927

Notes: Robust standard errors are in parentheses. [a] $p < 0.01$, [b] $p < 0.05$, [c] $p < 0.10$.

strategy of limiting the analysis to areas right outside the regulation boundaries to mitigate endogeneity concerns driven by unobserved differences between areas.

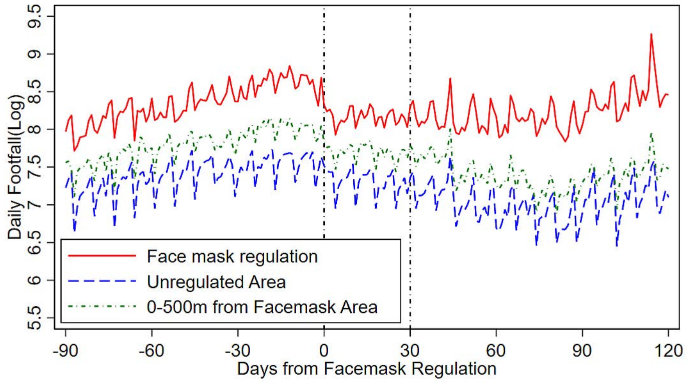

Fig S1. Outdoor facemask regulation and footfall
Notes: We show the log of footfall 90 days before and after facemask regulation for areas inside, outside and within 0-500m from regulated areas.

In Panel A and B in Figure S2 we plot the natural logarithm of daily footfall 90 days before and after various events and we control for a *fourth-order* polynomial (instead of a quadratic polynomial) on both sides of the threshold. Similar to Figure 4, we document a stark discontinuous drop in footfall after the 1st and 2nd lockdown is enforced. These results illustrate that the discontinuous drop in the footfall after the lockdowns are enforced is not sensitive to the specification of the time trend.

In Panel C in Figure S2 we investigate the effects of the partial lockdown. It is very clear that we do not observe any discontinuity in footfall around the implementation of the partial lockdown. We think this is not too surprising, as shops were still allowed to be open during the partial lockdown. Only part of the FNB sector (such as restaurants and pubs) was forced to cease operation while fast-food stores and 'on-the-go' food stores were still allowed to be open.

Panel D shows no jump in footfall after the 1st lockdown is relaxed. This makes sense as many shops remained closed for a while right after the 1st lockdown. However, it is evident that footfall increased over time, as shops started to reopen when lockdown restrictions were relaxed. Imperceptible changes in footfall are also recorded around the implementation of partial lockdown and facemask regulations (on the 30th September and the 1st of December, respectively). Hence it seems that these events were not stringent enough to restrict shopping mobility. We also estimate the impact of these events on footfall using difference-in-differences specifications. Similar to Figure S2, we do not record a discernible change in footfall around the partial lockdown, the removal of the 1st lockdown and the enforcement of facemask regulation on the 30th of September and the 1st December of 2020. These results are available upon request.

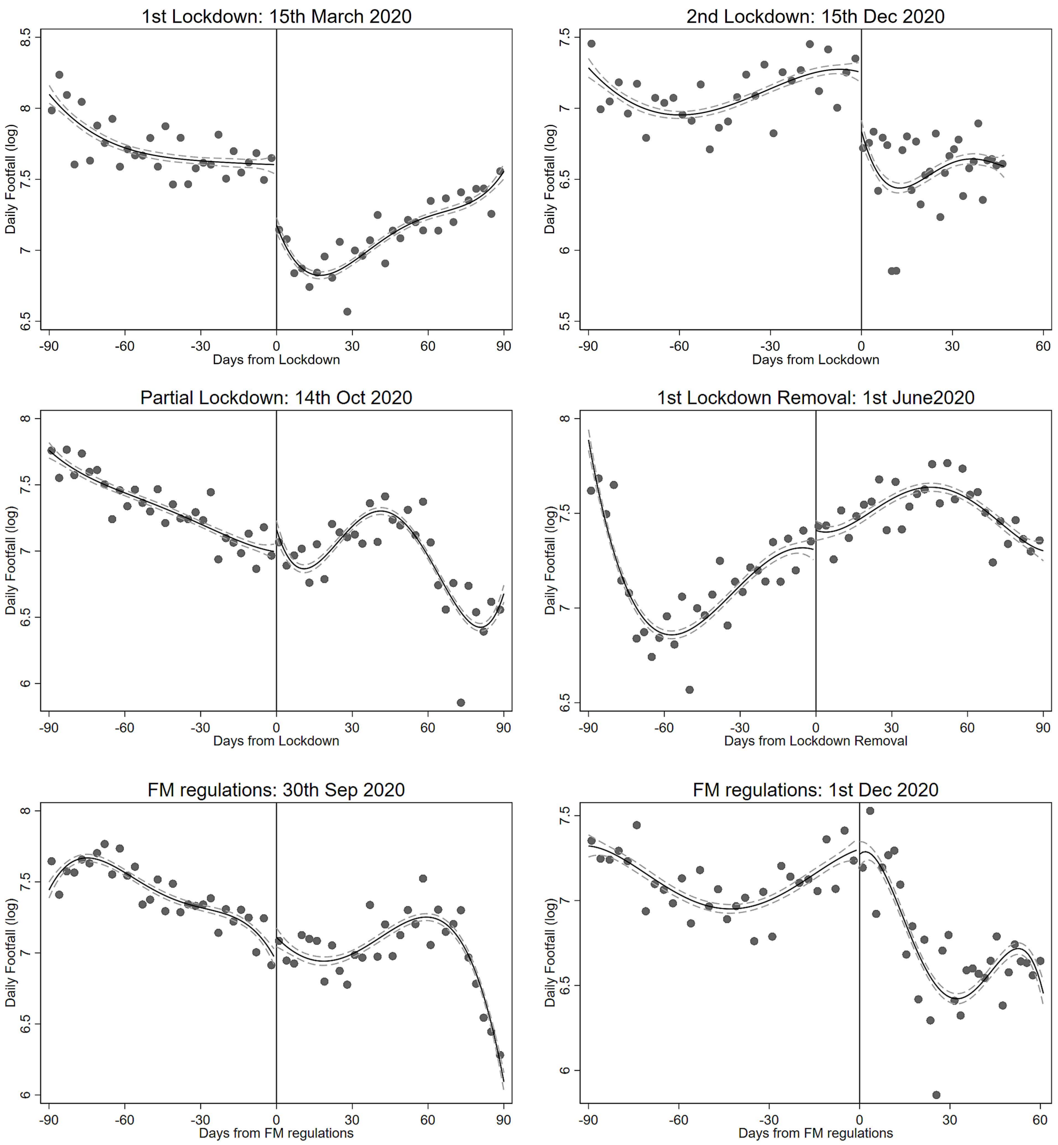

Fig S2. Lockdowns, Facemask Regulations and footfall

Notes: We show the log daily footfall 90 days before and after various events. We employ an Epanechnikov kernel and control for a fourth-order polynomial on both sides of the threshold. Dashed line denotes 95% confidence interval associated with fourth-order polynomial footfall trends.

Additional regression results

Table S6 presents baseline results of lockdowns on footfall from alternate specifications related to the Regression Discontinuity Design (RDD). In columns (1) to (4), we include first to fourth order polynomials of time trends before trimming down the sample to observations 60 and 30 days from the lockdowns respectively in columns (5) and (6). The rationale is to test whether baseline estimates in Table 2 holds under alternative specifications. It is comforting to observe that the estimates remain fairly robust in size and statistical significance across specifications and are comparable to main findings we report in Table 2, suggesting that our main findings are robust to other specifications and time windows.

Table S6. Effects of lockdowns on footfall (alternate specifications)

Polynomials/Event Window	**(1) First**	**(2) Second**	**(3) Third**	**(4) Fourth**	**(5) 60days**	**(6) 30days**
**Lockdowns**	-0.653^{a}	-0.635^{a}	-0.580^{a}	-0.646^{a}	-0.640^{a}	-0.647^{a}
	(0.081)	(0.078)	(0.085)	(0.106)	(0.077)	(0.102)
Obs	91822	91822	91822	91822	66773	35981
Adj R2	0.78	0.79	0.79	0.79	0.78	0.78

Notes: The dependent variable is the natural log of visitor footfall at RMC sensor i on day t. Reported variables denote binary variables that take the value of 1 (Lockdowns) for RMC sensors during the 1st Lockdown (from the 15th of March until the 1st of June 2020) and the 2nd Lockdown (from the 15th of December 2020 onwards). All regressions include RMC fixed effects and time trends (days to event) at various orders of polynomial as denoted by column headers. In (1)-(4), we estimate the effects of various events using single (linear) to fourth order polynomials of time trends. In (5) and (6), we further restrict the analysis to 60 and 30 days to a lockdown after controlling for second order polynomials of time trends (quadratic). Two-way clustered standard at postcode and date levels are reported in the parentheses. p<0.10, ^{b} p<0.05, ^{a} p<0.01.

Panels A and B of Table S7 present the stratified effects of the first and second lockdown on footfall separately. These results inform us on whether the effects associated with the 1st and 2nd lockdown on footfall are different from one another. As observed, we report a substantial drop in footfall after the 1st and 2nd lockdown are enforced and the estimated effects are quite comparable in size between the two events, and also similar to those reported in Table 2. Additional regressions suggest that density of shops matter as the effects of lockdowns are much greater along streets with a higher shop concentration. These results remain robust even we instrument shop counts with historical counts of cinema within 500m. These findings are again quite similar to those reported in Table 2.

Panels A and B of Table S8 repeat our analysis in Table 2, but we now examine the impact of shop counts and types for shops within 200m from the RMC sensors. The concern is whether computing shop counts and types within 500m is too broad. As observed, our reported estimates are quite similar to our initial findings reported in Table S8 in terms of size and direction. In Panel C, we repeat the analysis of Panel A in Table S8 but we further control for the daily number of hospital admissions due to Covid-19. We exclude this variable from our main analysis due to the concern that this variable could be a 'bad control', but we observe that the inclusion does not matter much to our estimates. If anything, the estimated effects of lockdowns on footfall are slightly smaller but they remain statistically significant. This is expected given that we do not expect much changes in the number of Covid-19 hospital admissions around the enforcement of the lockdowns.

Table S7. Effects of 1st and 2nd lockdowns on footfall

	**Panel A: 1st Lockdown**				
	**(1)**	**(2)**	**(3)**	**(4)**	**(5)**
	**Baseline**	**Shop Count**	**Shop Type**	**Combined**	**IV**
**1st Lockdown**	-0.728^{a} (0.145)	-0.714^{a} (0.140)	-0.730^{a} (0.143)	-0.725^{a} (0.141)	-0.722^{a} (0.141)
**1st Lockdown × Log Shop Counts**		-0.248^{a} (0.038)		-0.226^{a} (0.055)	-0.379^{a} (0.131)
**1st Lockdown × Share of Other Retail Shops**			-0.400 (0.359)	0.597 (0.452)	1.274^{c} (0.737)
**1st Lockdown × Share of Clothing Shops**			-2.773^{a} (0.463)	0.102 (0.778)	2.056 (1.743)
**1st Lockdown × Share of FNB shops**			-1.399^{b} (0.641)	-0.411 (0.518)	0.261 (0.737)
Obs	49620	49258	49258	49258	49258
Adj R2	0.81	0.82	0.82	0.82	0.41
Kleibergen-Paap F statistic					15.28
	**Panel B: 2nd Lockdown**				
	**(6)**	**(7)**	**(8)**	**(9)**	**(10)**
	**Baseline**	**Shop Count**	**Shop Type**	**Combined**	**IV**
**2nd Lockdown**	-0.708^{a} (0.122)	-0.699^{a} (0.122)	-0.692^{a} (0.123)	-0.688^{a} (0.122)	-0.673^{a} (0.122)
**2nd Lockdown × Log Shop Counts**		-0.138^{a} (0.036)		-0.074 (0.053)	-0.356^{a} (0.116)
**2nd Lockdown × Share of Other Retail Shops**			-3.132^{a} (0.496)	-2.787^{a} (0.565)	-1.466^{c} (0.822)
**2nd Lockdown × Share of Clothing Shops**			-3.981^{a} (0.567)	-3.032^{a} (0.836)	0.602 (1.605)
**2nd Lockdown × Share of FNB shops**			-2.375^{a} (0.532)	-2.019^{a} (0.534)	-0.656 (1.006)
Obs	42202	41515	41515	41515	41515
Adj R2	0.80	0.79	0.79	0.79	0.25
Kleibergen-Paap F statistic					14.84

Notes: The dependent variable is the natural log of visitor footfall at RMC sensor i on day t. Reported variables denote binary variables that take the value of 1 (1st Lockdown) for RMC sensors during the 1st Lockdown (from the 15th of March until the 1st of June 2020) for Panel A, and denote binary variables that take the value of 1 (2nd Lockdown) for RMC sensors after the 2nd Lockdown (from the 15th of December 2020 onwards) for Panel B. All regressions control for public and school holidays, weather conditions, RMC fixed effects and time trends (days to event) at second order polynomial of time trends (quadratic). We further restrict the analysis to a window 90 days from the event. In columns 5 and 10, we repeat the analysis in columns 4 and 9 (for all Panels) but we instrument log shop counts with the counts of historical cinemas in 1930. Two-way clustered standard at postcode and date levels are reported in the parentheses. ^{c} p<0.10, ^{b} p<0.05, ^{a} p<0.01.

Table S8. Effects of lockdowns and social distancing on footfall: robustness

	Panel A: Lockdowns(RDD) (200m)				
	**(1)**	**(2)**	**(3)**	**(4)**	**(5)**
	**Baseline**	**Shop Count**	**Shop Type**	**Combined(OLS)**	**Combined (IV)**
**Lockdowns**	-0.635[a]	-0.618[a]	-0.628[a]	-0.623[a]	-0.609[a]
	(0.078)	(0.073)	(0.076)	(0.073)	(0.076)
**Lockdowns × Log Shop Counts**		-0.179[a]		-0.121[a]	-0.411
		(0.032)		(0.045)	(0.372)
**Lockdowns × Share of Other Retail Shops**			-1.173[a]	-0.973[a]	-0.495
			(0.203)	(0.237)	(0.656)
**Lockdowns × Share of Clothing Shops**			-2.314[a]	-1.479[a]	0.522
			(0.259)	(0.370)	(2.628)
**Lockdowns × Share of FNB shops**			-1.241[a]	-1.098[a]	-0.757
			(0.253)	(0.231)	(0.566)
Obs	91822	90456	90456	90456	90456
Adj R2	0.79	0.78	0.78	0.78	0.35
Kleibergen-Paap F statistic					3.98
	**Panel B: Social Distancing(DID) (200m)**				
	**(6)**	**(7)**	**(8)**	**(9)**	**(10)**
	**Baseline**	**Shop Count**	**Shop Type**	**Combined(OLS)**	**Combined (IV)**
**Social Distancing**	-0.626[a]	-0.621[a]	-0.631[a]	-0.627[a]	-0.629[a]
	(0.034)	(0.034)	(0.038)	(0.034)	(0.038)
**Social Distancing × Log Shop Counts**		-0.091[b]		-0.149[b]	-0.071
		(0.039)		(0.061)	(0.188)
**Social Distancing × Share of Other Retail Shops**			-0.186	0.066	-0.066
			(0.244)	(0.321)	(0.367)
**Social Distancing × Share of Clothing Shops**			-0.484[b]	0.547	0.007
			(0.189)	(0.448)	(1.342)
**Social Distancing × Share of FNB shops**			-0.361	-0.192	-0.281
			(0.375)	(0.344)	(0.459)
Obs	234152	232689	232689	232689	232689
Adj R2	0.85	0.85	0.85	0.85	0.21
Kleibergen-Paap F statistic					3.77
	**Panel C: Lockdowns (RDD) (Control for Covid-19 Hospital Admissions)**				
	**(11)**	**(12)**	**(13)**	**(14)**	**(15)**
	**Baseline**	**Shop Count**	**Shop Type**	**Combined(OLS)**	**Combined (IV)**
**Lockdowns**	-0.620[a]	-0.607[a]	-0.613[a]	-0.607[a]	-0.595[a]
	(0.081)	(0.076)	(0.079)	(0.076)	(0.076)
**Lockdowns × Log Shop Counts**		-0.155[a]		-0.114[b]	-0.362[a]
		(0.029)		(0.047)	(0.111)
**Lockdowns × Share of Other Retail Shops**			-1.406[a]	-0.881[c]	0.268
			(0.374)	(0.467)	(0.757)
**Lockdowns × Share of Clothing Shops**			-2.746[a]	-1.287[c]	1.904
			(0.413)	(0.698)	(1.516)
**Lockdowns × Share of FNB shops**			-1.397[a]	-0.864[b]	0.300
			(0.450)	(0.411)	(0.786)
Obs	72095	71192	71192	71192	71192
Adj R2	0.78	0.77	0.77	0.77	0.24
Kleibergen-Paap F statistic					14.91

Notes: The dependent variable is the natural log of visitor footfall at RMC sensor i on day t. Reported variables denote binary variables that take the value of 1 (Lockdowns) for RMC sensors during the 1st Lockdown (from the 15th of March until the 1st of June 2020) and 2nd Lockdown (from the 15th of December 2020 onwards) for Panel A. RDD regressions from columns (1) to (5) in Panel A include controls for public and school holidays, weather conditions, RMC fixed effects and time trends (days to event) at second order polynomial of time trends (quadratic). We further restrict the analysis to a window 90 days from the event. Reported variables denote binary variables that take the value of 1 (Social Distancing) for RMC sensors after the social distancing is enforced (from the 1st of June 2020 to the 13th of October 2020) for Panel B. DID regressions from columns (6) to (10) in Panel B include RMC fixed effects, day-of-week fixed effects (Monday to Sunday) and week fixed effects (1-52 weeks) and year fixed effects. Baseline effects of lockdowns and social distancing are reported in columns (1) and (5) respectively. In columns (2) and (7), we further interact these binary variables with the demean natural logarithm of shop counts within 200m from the RMC. In columns (3) and (8), we interact binary event variables with demean share of shops (whether Daily shopping, Clothing, Food and Beverages(FNB) or other retail) within 200m from the RMC. In columns (4) and (9), we collectively estimate how shop counts and shop types can affect the impacts of lockdowns and social distancing on footfall. In columns (5) and (10), we repeat the analysis in columns (4) and (9) but we instrument log shop counts with the counts of historical cinemas in 1930. RDD regressions from columns (11) to (15) in Panel C is similar to Panel A of Table 2 but we further control for daily Covid-19 hospitalizations. Two-way clustered standard at postcode and date levels are reported in the parentheses. [c] $p<0.10$, [b] $p<0.05$, [a] $p<0.01$.
